# Host Chemical Footprints Induce Host Sex Discrimination Ability in Egg Parasitoids

**DOI:** 10.1371/journal.pone.0079054

**Published:** 2013-11-11

**Authors:** Ezio Peri, Francesca Frati, Gianandrea Salerno, Eric Conti, Stefano Colazza

**Affiliations:** 1 Dipartimento di Scienze Agrarie e Forestali, Università degli Studi di Palermo, Palermo, Italy; 2 Dipartimento di Scienze Agrarie e Ambientali, Università degli Studi di Perugia, Perugia, Italy; University of California, Berkeley, United States of America

## Abstract

*Trissolcus* egg parasitoids, when perceiving the chemical footprints left on a substrate by pentatomid host bugs, adopt a motivated searching behaviour characterized by longer searching time on patches were signals are present. Once in contact with host chemical footprints, *Trissolcus* wasps search longer on traces left by associated hosts rather than non-associated species, and, in the former case, they search longer on traces left by females than males. Based on these evidences, we hypothesized that only associated hosts induce the ability to discriminate host sex in wasps. To test this hypothesis we investigated the ability of *Trissolcus basalis*, *T. brochymenae,* and *Trissolcus* sp. to distinguish female from male *Nezara viridula*, *Murgantia histrionica*, and *Graphosoma semipunctatum* footprints. These three pentatomid bugs were selected according to variable association levels. Bioassays were conducted on filter paper sheets, and on *Brassica oleracea* (broccoli) leaves. The results confirmed our hypothesis showing that wasps spent significantly more time on female rather than male traces left by associated hosts on both substrates. No differences were observed in the presence of traces left by non-associated hosts. The ecological consequences for parasitoid host location behaviour are discussed.

## Introduction

Successful reproduction of insect parasitoids is linked to adult female behavioural decisions that lead them to find suitable hosts often living in highly complex environments [1]. During their foraging behaviour, parasitoids rely on a series of visual, tactile and chemical stimuli, although the chemical cues play the major role [2]–[5]. Wasps use volatile compounds from the plant or host/plant complex to locate a suitable host habitat at long distance, and low volatile compounds for host location at short distance and for host recognition and acceptance. When wasps land on a plant, they can taste chemical traces left by herbivores walking over the leaves as direct or indirect cues, leading them to the host targets [6]–[11]. Chemical footprints left by larvae of *Spodoptera frugiperda* (JE Smith) (Lepidoptera: Noctuidae) directly drive the larval parasitoid *Cotesia marginiventris* (Cresson) [6], while pentatomid adult chemical footprints indirectly drive their platygastrid egg parasitoids [12]–[14]. Host eggs are generally available for a short period, leaving egg parasitoids a limited window of opportunity to exploit them [3], [15]. Therefore, indirect host related cues, such as adult footprints, represent reliable cues for egg parasitoids to optimize energy and time by restraining their search to areas where newly laid host eggs are likely to be found [3], [4], [16], [17]. In the field, however, host plants could be infested by several phtytophagous species and thus contaminated by a plethora of chemical traces. Consequently, platygastrid wasps have developed the ability to discriminate between footprints left by true bugs at different association levels, i.e. associated and non-associated species, the latter being for example occasional hosts attacked in the field or factitious hosts used in laboratory, but also species that elicit parasitoid responses but are not suitable for parasitoid development [18], [19]. In this context, the response of platygastrid wasps to host chemical footprints left by pentatomid adults represents an example of these host-parasitoid interactions. *Trissolcus simoni* (Mayr) searches longer on chemical trails left by its associated host, *Eurydema ventrale* Kolenati, and responds weakly to contact cues of the non-associated species *Murgantia histrionica* (Hahn) and *Nezara viridula* (L.). Similarly, *Trissolcus brochymenae* (Ashmead) strongly responds to chemical trails left by its associated hosts, *M. histrionica*, and weakly to footprints from the non-associated species, *E. ventrale* and *N. viridula* [18]. Since host female traces are the most promising signals of host eggs, platygastrid females have enhanced this strategy and distinguish between footprints left by females and males of their associated hosts [12], [18], [20], [21]. In this scenario, we hypothesized that host sex discrimination ability was strictly related to host specificity. In other words, the wasp ability to distinguish male and female footprints has evolved only to find associated hosts, as wasps only invest resources to obtain a reward. Moreover, the strategy of host footprints exploitation could be further modulated by egg parasitoids dietary specialization. In fact, according to the concept of host range and infochemical use in natural enemies, specialist species use specific cues more frequently than generalist ones [4], [22].

To test this hypothesis, we examined under laboratory conditions the ability of three platygastrid egg parasitoid species to detect adult host sex on the basis of the host traces left by three pentatomid species on natural and artificial substrates. The egg parasitoids, *Trissolcus basalis* (Wollaston), *T. brochymenae* and *Trissolcus* sp., were selected according to their dietary specialization, considering *T. basalis* as ‘generalist at the host and the host plant/feeding substrate levels’, and *T. brochymenae* as ‘specialist at host/prey and nearly at host plant/feeding substrate level’ [4], [22]. The pentatomid hosts *N. viridula*, *M. histrionica*, and *Graphosoma semipunctatum* (F.) were selected according to specificity of the host-parasitoid relations (Table 1). The substrates for host traces were filter paper and *Brassica oleracea* L. (broccoli) leaves. Filter paper was selected to avoid possible confounding or masking effects of leaf morphological or chemical features on phytophagous and parasitoid behaviour. Filter paper does not interfere with parasitoid behaviours or with the chemical properties of host cues allowing to obtain similar results as in natural conditions. Broccoli leaves were selected because they represent a natural substrate for two of the three pentatomid species used in the experiments, *N. viridula* and *M. histrionica*.

**Table 1:** Field and laboratory relationships among host bugs and egg parasitoid species used in the experiments.

			Pentatomid hosts			
			*Graphosoma semipunctatum*	*Murgantia histrionica*		*Nezara viridula*
	***Trissolcus*** ** sp.**		• sympatric, associated species	–		–
**wasps**	***Trissolcus brochymenae***		• **in field**: allopatric, no association			• **in field:** allopatric, no association [23].
**Platygastrid**	specialist at host/prey andnearly at host plant/feeding substratelevel [4], [22]		• **in laboratory**: wasp does notaccept eggs	• sympatric, associated species		• **in laboratory**: wasp responds to host volatile and contact cues; recognizes and accepts eggs; does not emerge [18]
	***Trissolcus basalis***		• **in field**: sympatric, no recorded association	• **in field**: allopatric, noassociation [24]		
	generalist at the host and the host plant/feeding substrate levels [4], [22]	• **in laboratory**: wasp does notrespond to host volatiles; respondsto host chemical trails; recognizesand accepts eggs, and emerges [19]		• **in laboratory**: wasp does not respond to host volatiles, responds to host chemical trails, recognizes and accepts eggs; does not emerge [19]	• sympatric, associated species	

## Materials and Methods

### Insects

All pentatomid species were reared in climate rooms (25±1°C, 60±5% RH, L16:D8) inside plastic cages (30×19.5×12.5 cm) with 5 cm diameter mesh-covered holes. All stages were fed with their preferred food. Seeds of wild *Ferula communis* L. were collected for *G. semipunctatum*, vegetative parts of broccoli (*B. oleracea*) were fed to *M. histrionica*, and sunflower seeds (*Helianthus annus* L.) and French beans (*Phaseolus vulgaris* L.) to *N. viridula.* No specific permits were required for collection of insects. The collection sites were not privately owned or protected in any way and field samplings did not involve endangered or protected species. Newly laid egg masses were transferred to other cages for nymph development. Every 2–3 d, single nymphs of the last instar were gently transferred, using a thin brush, to single plastic pots (Ø = 40 mm, h = 65 mm). Nymphs were checked daily until adult emergence, so that individuals were of known age and were available for experiments. Bugs used for bioassay preparation were adult males and females. Mated adults were obtained from pairs that had copulated. They were separated immediately after mating and isolated individually for 24 h before the experiment. Adults used for the bioassays were approximately 10–14 days post-emergence, with females in pre-ovipositional physiological state.

Wasp species were reared in 85-ml glass tubes, fed with a Safavi sugar-water diet [25], and maintained at controlled conditions (25±1°C, 60±10% RU and L16:D8). Two-three times *per* week, 1-2-day-old egg masses of *M. histrionica, N. viridula* and *G. semipunctatum* were exposed to *T. brochymenae, T. basalis* and *Trissolcus sp.*, respectively. Parasitized egg masses were kept in the same environmental conditions described above until the emergence of adult wasps. After emergence, wasp males and females were kept together to allow mating. Females used for the bioassays were 2 to 3 days old, and naïve to oviposition experience and contact with host chemical traces. About 16–17 h before the bioassays, they were individually isolated in 2-ml glass vials provided of a drop of Safavi sugar-water diet; vials were closed with a cotton plug. Female wasps were transferred to the bioassay room (25±1°C, 50±10% RH) to acclimatize at least 30 min before bioassays.

### Experiment 1: Parasitoid Response to Host Chemical Footprints on Filter Paper

The experiment was conducted in an open arena consisting of a square sheet of filter paper (25×25 cm; wasp/arena ratio: 0.003%). In the centre of the arena, a circular area (6·cm diameter; 28.26 cm^2^, about 4.5% of the entire arena; wasp/arena ratio: 0.071%), defined by a cardboard mask put on the filter paper, was exposed for 30·min to a single, male or female, adult bug, while the surrounding area was left uncontaminated. To ensure bug legs were in constant contact with the filter paper and, at the same time, to avoid surface contamination with bug volatiles, adults were constrained under a steel mesh cover (6·cm diameter, 1·cm high, 0.01·cm mesh) and forced to walk with a special device [18]. Open arenas contaminated by bug’s faeces were not used for bioassays. Each parasitoid species was tested on traces left by male and female adults of the three pentatomid species. For each treatment 25 *T. basalis* females, 25 *T. brochymenae* females and 15 *Trissolcus* sp. females were tested with a total of 390 wasps. The trial was stopped when the female flew away or walked off the whole arena. The arrestment responses of female wasps were measured as residence time in the entire arena, i.e. pooling time spent by wasps inside and outside the circular contaminated area.

### Experiment 2: Parasitoid Response to Host Chemical Footprints on Broccoli Leaves

Parasitoids’ response to leaf surface contaminated by host footprints was investigated in an open arena consisting of a leaf disk adaxial surface (5-cm in diameter; wasp/arena ratio: 0.102%), cut out from a fully expanded broccoli leaf. Plants were obtained from certified seed material (Esasem S.p.a., Casaleone -VR- Italy), grown individually in 14-cm plastic pots filled with fertilized commercial soil (Terflor - HOCHMOOR), in greenhouse conditions (20±2°C, 60±5% RH, 12∶12 L:D), and watered daily. Four-week-old plants with 5–6 fully expanded leaves were used for the experiments. The cut leaf petiole was wrapped in wet cotton and inserted in a 1-ml vial filled with distilled water and sealed with Parafilm®. A male or female bug was allowed to walk over the disk adaxial surface and produce chemical footprints according to the method described in the previous experiment. After 1 h treated leaf disks were cut with a razor and used for the behavioural assays. Mated adults with excised stylets were used to prevent bug feeding and to obtain leaf disks with chemical traces. For stylet excision, bugs were previously anaesthetized inside a glass tube with CO_2_ for 4–5 s in order to immobilize their labium. Stylets were drawn from labium using an entomological pin (no. 000) and were amputated half their length using precision micro-scissors under a stereomicroscope (Zeiss Stemi SV8) with optical fibre illumination (Intralux 5000). Bugs were then placed inside a plastic dish (12 cm diameter) for 24 h allowing them to recover, and were subsequently used to contaminate the cabbage leaf disk as described above. Wasp and arena were discarded after each successful bioassay. For each treatment 25 *T. basalis* females and 25 *T. brochymenae* females were tested on traces left by male and female adults of the three pentatomid species, for a total of 300 wasps. *Trissolcus* sp. females were not tested because broccoli plant - *G. semipunctatum* - *Trissolcus* sp. is not a natural association. The trial was stopped when the female wasp flew away or reached the disk edge. The wasp arrestment response was measured as residence time spent on the leaf adaxial surface.

### Video Tracking and Motion Analysis

The arena was illuminated from above by two 22-W cool white fluorescent tubes (Full spectrum 5900 K, 11W; Lival, Italy). Wasp females were gently released singly into the centre of the treated area. Wasps that immediately displayed the typical arrestment posture, i.e. motionless with the antennae in contact with the leaf surface were scored as “responding”. Wasps that did not show the arrestment response were recaptured and retested approximately 1 min later. After three unsuccessful trials, wasps were considered “non-responding” and excluded from the data analysis. Responding female behaviour was recorded using a monochrome CCD video camera (Sony SSC M370 CE) fitted with a 12.5–75 mm/F 1.8 zoom lens. Analog video signals from the camera were digitized by a video frame grabber (Studio PCTV–Pinnacle Systems, Mountain View, CA). Digitalized data were processed by XBug, a video tracking and motion analysis software [26]. The trial was stopped when the female flew away or walked off the paper arena or the leaf disk. Wasp and arena were discarded after each successful bioassay. Tests were conducted from 8∶30 to 14∶00 h. The bioassay room temperature was 26±1°C.

### Statistical Analyses

Residence times (s) of parasitoid wasps on arenas with male and female host footprints were compared using Student’s *t-test* for independent samples. Statistical analyses were processed using Statistica7 software [27]. Data were transformed using the logarithmic function before the analyses [28].

## Results

### Experiment 1: Parasitoid Response to Host Chemical Footprints on Artificial Substrate

**Figure 1:**
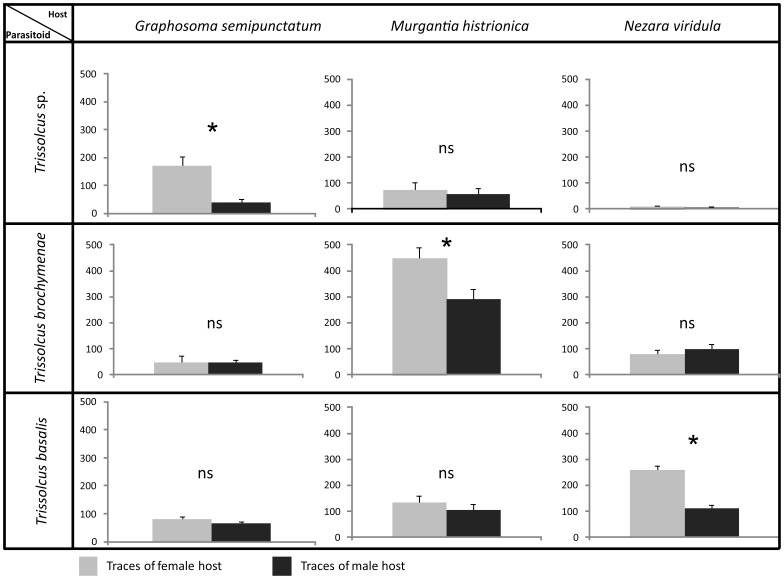
Host sex discrimination ability of *Trissolcus* females exploring an artificial substrate contaminated with bug chemical footprints. Searching time of females of three *Trissolcus* species exploring an artificial substrate contaminated with chemical footprints laid by adult males and females of three pentatomid species. The number of tested wasp females was 15 for *Trissolcus* sp. and 25 each for *T. basalis* and *T. brochymenae*. Bars indicate the duration (means ± SE) of the residence time of wasp females. Asterisks (*) indicate p<0.05 by Student’s *t-test* for independent samples. NS, not significant.

The percentage of wasp females that responded during the bioassays ranged from 80 to 100. The response of wasp females to chemical footprints left on the artificial substrate by male and female associated and non-associated adult pentatomids is illustrated in Figure 1. Naïve wasp females discriminated between chemical traces left by a pentatomid female versus male, exhibiting a clear preference for female traces only when these belonged to their associated hosts. In details, *Trissolcus* sp. females spent more time in arenas contaminated by bug females than males when in contact with traces left by its associated host, *G. semipunctatum,* (t = 2.46, *df* = 27, *P* = 0.021). No significant differences were observed when wasps were in contact with footprints of non-associated species (*M. histrionica:* t = 0.31, *df* = 28, *P* = 0.761, and *N. viridula:* t = 0.48, *df* = 28, *P* = 0.635). Similarly, *T. brochymenae* wasps encountering traces of its associated host, *M. histrionica*, showed longer arena residence time on female versus male residues (t = 3.13, *df* = 40, *P* = 0.003). No differences emerged when the arena was contaminated by non-associated species (*G. semipunctatum:* t = −0.59, *df* = 46, *P* = 0.558; and *N. viridula:* t = 0.043, *df* = 46, *P* = 0.966). Finally, arena residence time of *T. basalis* females was significantly higher on chemical footprints left by *N. viridula* females versus *N. viridula* males (t = 6.82, *df* = 48, *P*<0.001). No significant differences were observed when the wasps were in contact with footprints of non-associated species (*G. semipunctatum*: t = 1.22, *df* = 48, *P* = 0.229; and *M. histrionica*: t = 1.38, *df* = 48, *P* = 0.175).

### Experiment 2: Parasitoid Response to Host Chemical Footprints on Leaf Disks

**Figure 2:**
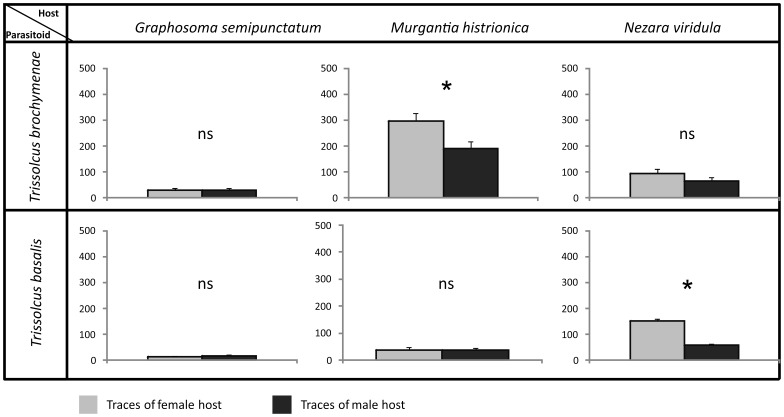
Host sex discrimination ability of *Trissolcus* females exploring a natural substrate contaminated with bug chemical footprints. Searching time of females of two *Trissolcus* species exploring the adaxial surface of a disk from broccoli leaf, contaminated with chemical footprints laid by adult males and females of three pentatomid species. The number of tested wasp females was 30 for each treatment. Bars indicate the duration (means ± SE) of the residence time of wasp females. Asterisks (*) indicate p<0.05 by Student’s *t-test* for independent samples. NS, not significant.

The percentage of wasp females that responded in bioassays ranged from 76 to 100. However, when *T. basalis* was tested on *G semipunctatum* traces, responding females dropped to 32% on female traces and to 24% on male traces. The response of *T. basalis* and *T. brochymenae* females to chemical footprints left on the leaf surface by male and female of associated and non-associated species is illustrated in Figure 2. As in experiment 1, both wasp species were able to discriminate between chemical traces left on leaf disks by host female versus male only when testing associated hosts. In fact, *T. brochymenae* females showed significantly longer residence time on leaf disks contaminated by chemical traces of *M. histrionica* females versus males, (t = 3.11, *df* = 48, *P = *0.003), but not on disks contaminated by female of non-associated species (*G. semipunctatum:* t = −0.29, *df* = 48, *P* = 0.774; and *N. viridula:* t = 1.29, *df* = 48, *P* = 0.204). Analogous results are obtained for *T. basalis* females which spent more time in the arena contaminated by *N. viridula* females versus males (t = 12.35, *df* = 41, *P* = 0.001), whereas no significant differences emerged in responses to females and males of non-associated species (*G. semipunctatum:* t = −0.40, *df* = 12, *P* = 0.70; and *M. histrionica*: t = 0.06, *df* = 44, *P* = 0.95).

## Discussion

*Trissolcus* egg parasitoids were shown to respond positively to the footprints of females of those host species they are normally associated with. In contrast, no positive response was observed to footprints of males of either species or footprints of females of pentatomid species they are not normally associated with (Table 1). Thus, our study shows that *Trissolcus* egg parasitoids have developed the ability of host sex discrimination only for those species they are highly adapted to.

In general, the response of wasps to host chemical traces was not influenced by the substrate used in the bioassays. However, the low percentage of *T. basalis* females responding to chemical footprints left by *G. semipunctatum* on broccoli leaves shows that plant surfaces can modulate the host/parasitoid relation, as they adsorb and release host contact kairomones [6], [7], [9], [11]. Different epicuticular wax compositions and/or the presence of leaf morphological features such as trichomes and veins [29]–[31] might also interfere with the attachment and mobility of insect herbivores and natural enemies [32]–[35].

Chemical footprints represent, for *Trissolcus* species, indirect host related contact cues, that induce wasp females to search longer (“motivated searching” [3]) on host patches where such cues are present, and to reinforce response by systematically returning to stimuli after losing contact [17]. If not rewarded by successful oviposition, wasps gradually lose their motivated searching response and regress to general host searching behaviour, as reported for *T. basalis* on *N. viridula* traces [36]. Therefore, egg parasitoids spy on host footprints to restrict searching to an area where host eggs are more likely to be found. Host eggs are generally available during a short time due to their rapid development [3]. Thus, egg parasitoid ability to distinguish between footprints left by host and non host bugs, and discriminate between male and female traces, allows them to modulate host search behaviour, spying more reliable cues for host eggs, and avoid following ‘false leads’ and wasting time and energy searching patches devoid of hosts [37]–[39]. The behaviour adopted by *Trissolcus* wasps is a good example of this strategy. In fact, detecting *n*-nonadecane, a sex-specific cuticular hydrocarbon from *N. viridula* males, allows *T. basalis* females to distinguish between male or female bug residues [20]. A more finely tuned strategy was developed by *T. brochymenae* females. To find newly laid eggs of its associated host, *M. histrionica*, they exploit cues that are strongly correlated with oviposition, since they are able to discriminate the footprints left by mated host females that have not yet laid eggs [21].

The strength of wasp female responses to chemical footprints left by associated hosts could be considered a step of a pairwise co-evolution of insect host-parasitoid associations shaped by the natural selection [3], [5]. At all trophic levels, organisms do not evolve independently. They co-evolve in an antagonistic or, more rarely, mutualistic way, reaching different levels of benefit and fitness trade-offs [40]. In the case of foraging parasitoids, it is possible to speculate co-evolutionary interactions within the pair, host and parasitoid. For example, wasps preferentially search for hosts in higher quality patches, and, as a co-evolutionary response, hosts lay eggs in poorer patches [41]. The *T. basalis* ability to discriminate between male and female host footprints could be an important strategy to localize the host egg masses of *N. viridula*, as this pest exhibits a tendency to lay egg masses far from sites where adults feed and mate [12].

Moreover, by adaptations to direct/indirect chemical cues from their host or from plant-host complex, like kairomones and synomones, co-evolution can drive parasitoids to specialization. Several examples of host specificity based on response to chemical cues from either herbivores or herbivore – host plant complexes have been reported. *Aphidius ervi* Haliday (Hymenoptera: Braconidae) discriminates between plants damaged by the pea aphid *Acyrthosiphon pisum* (Harris) (Homoptera: Aphididae) and non host *Aphis fabae* (Scop.) [42]. The larval parasitoid *Cardiochiles nigriceps* Viereck (Hymenoptera: Braconidae) is attracted only to host induced plant volatiles (HIPVs) emitted by different plant species that were damaged by its host *Heliothis virescens* (F.) (Lepidoptera: Noctuidae), whereas it does not respond to volatiles from the same plants species if they were attacked by *Helicoverpa zea* (Boddie) (Lepidoptera: Noctuidae) [43]. Similarly, specific kairomones emitted by phytophagous hosts themselves can allow wasp females to differentiate between host species [44]. For example, in their host location process *Tiphia vernalis* Rohwer and *Tiphia pygidialis* Allen (Hymenoptera: Tiphiidae), larval ecto-parasitoids of, respectively, the Japanese beetle, *Popillia japonica* Newman (Coleoptera: Scarabaeidae), and the masked chafer, *Cyclocephala* spp, (Coleoptera: Scarabaeidae) showed significant preference for compounds from the products of their hosts rather than non-hosts [45]. Meiners et al. [46] demonstrated that the egg parasitoid *Oomyzus gallerucae* Fonscolombe (Hymenoptera: Eulophidae) distinguishes between fecal kairomones of its host, the elm leaf beetle *Xanthogaleruca luteola* ( = *Pyrrhalta*) (Muller) (Coleoptera: Chrysomelidae), and non-host caterpillar, *Opisthograptis luteolata* L. (Lepidoptera: Geometridae). In the *Trissolcus* genus, host specificity was previously reported in terms of host chemical footprints exploitation. In a comparative laboratory analysis, *T. basalis* females showed a motivated search behaviour when in contact with chemical trails left on filter paper by three species of pentatomid bugs, e.g., *E. ventrale*, *M. histrionica* and *G. semipunctatum.* However, *T. basalis* response was less intense than in the presence of traces left by its associated host *N. viridula* [19]. Similarly, *T. simoni* and *T. brochymenae* partially respond to chemical footprints of different bugs, and discriminate footprints of their associated hosts, *E. ventrale* and *M. histrionica*, respectively, from those of non-associated species [18].

Thus, the use of host chemical traces by foraging *Trissolcus* females appears to be related to host specificity. This was confirmed by our results. *Trissolcus* sp, *T. basalis* and *T. brochymenae* perceive chemical traces of associated and non-associated species, but they show host sex discrimination ability only in the presence of chemical footprints from their associated host species. On the contrary, the ability to discriminate the host sex in non-associated species does not seem to be modulated by the dietary specialization, although different strategies to exploit host chemical footprints related to their different dietary specialization, have been shown in *T. basalis* and *T. brochymenae* when in contact with substrates contaminated by their associated hosts. In fact, as already discussed above, *T. brochymenae,* specialist at host/prey and nearly at host plant/feeding substrate level, is able to discriminate the chemical traces left by host females that had mated but had not yet laid host eggs from those left by virgin or parous host females. This parasitoid preference is strictly related to the transfer of sperm and associated substances from host males to females during copulation [21]. On the other hand, *T. basalis*, a generalist at the host and the host plant/feeding substrate level, prefers host female to male traces in all physiological conditions, i.e. virgin and mated [12].

Inability to discriminate host sex in non-associated hosts could be explained in terms of costs that insects should sustain to acquire the relevant information to tell the host apart from non-host, costs that are not acceptable when the information is poor [47].

Finally, although host range evolution is probably dynamic, with repeated host range expansions followed by re-specialization [48], our results provide new information evidencing that parasitoids’ host specificity, linked to host chemistry, limits the risks of non-target effects in biological control programs. The inability of *Trissolcus* species to discriminate the sex of non-associated species would reduce probability that they attack these pentatomid species in field. Therefore, as suggested by Conti et al. [18] and Salerno et al. [19], the development of “new associations” between *Trissolcus* wasps and non-associated pentatomid bugs (e.g. *N. viridula – T. brochymenae* and *G. semipunctatum - T. basalis*) appears unreliable under field conditions, due to parasitoid inability to exploit semiochemical cues.
